# Genetic polymorphisms of immune checkpoint proteins PD-1 and TIM-3 are associated with survival of patients with hepatitis B virus-related hepatocellular carcinoma

**DOI:** 10.18632/oncotarget.8435

**Published:** 2016-03-28

**Authors:** Zhu Li, Na Li, Fang Li, Zhihua Zhou, Jiao Sang, Zhao Jin, Huihui Liu, Qunying Han, Yi Lv, Zhengwen Liu

**Affiliations:** ^1^ Department of Infectious Diseases, First Affiliated Hospital of Xi'an Jiaotong University, Xi' an, 710061, Shaanxi, China; ^2^ Xi'an Medical University, Xi'an, 710021, Shaanxi, China; ^3^ Department of Hepatobiliary Surgery, First Affiliated Hospital of Xi'an Jiaotong University, Xi'an, 710061, Shaanxi, China; ^4^ Institute of Advanced Surgical Technology and Engineering, Xi'an Jiaotong University, Xi' an, 710061, Shaanxi, China

**Keywords:** hepatitis B virus, hepatocellular carcinoma, immune checkpoint, PD1, TIM3

## Abstract

Programmed cell death protein 1 (PD-1) and T-cell immunoglobulin domain and mucin domain containing molecule 3 (TIM-3) are involved in hepatitis B virus (HBV) infection and hepatocellular carcinoma (HCC). This study examined the associations of *PD1* and *TIM3* polymorphisms with the overall survival (OS) of a prospective cohort of 258 HBV-related HCC patients. Results showed that *PD1* +8669 G allele-containing genotypes or *TIM3* −1516 genotype GG were significantly associated with longer OS (*P* < 0.001 and *P* = 0.001, respectively). In multivariate analysis, *PD1* +8669 G allele-containing genotypes and *TIM3* −1516 genotype GG were independently associated with longer OS (hazard ratio (HR), 1.835; 95% confidence interval (CI), 1.342–2.509; *P* < 0.001 and HR, 2.070; 95%CI, 1.428–3.002; *P* < 0.001, respectively). *PD1* +8669 G allele-containing genotypes were significantly associated with longer OS in patients receiving surgical (resection or radiofrequency) treatment, transcatheter arterial chemoembolization (TACE) or supportive and symptomatic treatment. *TIM3* −1516 genotype GG was significantly associated with longer OS in TACE patients. In multivariate analysis, *PD1* +8669 G allele-containing genotypes were independently associated with longer OS in each treatment population. *TIM3* −1516 genotype GG was independently associated with longer OS in patients receiving surgical treatment or TACE. These findings suggest that *PD1* +8669 A/G and *TIM3* −1516 G/T polymorphisms may affect the prognosis of HBV-related HCC and may be new predictors of prognosis for HCC patients.

## INTRODUCTION

Hepatocellular carcinoma (HCC) is one of the most common cancers worldwide [1]. HCC is among the malignancies with high morbidities and mortalities in China and hepatitis B virus (HBV) infection is a predominant causative agent associated with the development of HCC [2, 3]. The deterioration of T cell function, a state termed T cell exhaustion, impairs the immunity to chronic viral infections including HBV infection and alters the immunology of cancer [4].

The underlying mechanisms of T cell exhaustion in chronic HBV infection and HBV-related HCC have not been fully clarified. However, programmed cell death protein 1 (PD-1) and T-cell immunoglobulin domain and mucin domain containing molecule 3 (TIM-3), two major immune checkpoint proteins, have been shown to be involved in the T-cell dysfunction and exhaustion of chronic HBV infection and HCC development [5–9]. PD-1 and TIM-3 expression has also been indicated to be related to the outcome and the disease progression of HBV infection [10–12] and the prognosis of HCC [9, 13]. Furthermore, blocking PD-1 or TIM-3 pathway restores antiviral T-cell responses in chronic HBV infection [6, 14, 15]. Genetically, polymorphisms of *PD1* and *TIM3* genes have been revealed to be associated with the disease progression and the development of HCC in chronic HBV infection [16–18].

However, whether the polymorphisms of *PD1* and *TIM3* are of clinical significance in the prognosis of HBV-related liver disease, especially HCC, remains unknown. Therefore, the aim of this study is to examine the associations of *PD1* and *TIM3* gene polymorphisms with the overall survival (OS) of patients in a prospective cohort of patients with HBV-related HCC receiving various treatments.

## RESULTS

### Characteristics of patients

The demographics and the clinical characteristics of the 262 HBV-related HCC patients at study entry and the treatments were summarized in Table 1. The median follow-up period was 32.0 (3.0–77.0) months for the whole study population.

**Table 1:** Demographics and clinical features of the 262 HBV-related HCC patients at study entry

Variables	Results
Age (median (range)) y	50.00 (19.00–77.00)
Gender (male/female)	230/32
HBV DNA (logIU/mL)	3.97 ± 1.38
ALT (IU/L, median (range))	46.00 (5.00–970.00)
AST (IU/L, median (range))	59.50 (6.50–1320.00)
TBIL (μmol/L, median (range))	23.80 (1.35–763.15)
ALB (g/L, median (range))	34.10 (19.30–54.20)
AFP (< 200/≥ 200, n)	145/117
Child-Pugh score (A/B/C, *n*)	164/51/47
Tumor size (< 5 cm/≥ 5 cm, *n*)	174/88
TNM stage (I/II/III/IV, *n*)	75/83/91/13
Treatments (LT/SR/RF/TACE/ST/SS, *n*)	2/52/55/74/2/77

Abbreviations: HCC, hepatocellular carcinoma; HBV, hepatitis B virus; ALT, alanine aminotransferase; AST, aspartate aminotransferase; TBIL, total bilirubin; ALB, albumin; AFP, α-fetoprotein; LT, liver transplantation; SR, surgical resection; RF, radiofrequency ablation; TACE, transcatheter arterial chemoembolization. ST, sorafenib treatment; SS, supportive and symptomatic treatment.

### Association of *PD1* and *TIM3* polymorphisms with overall survival of HBV-related HCC patients

**Figure 1:**
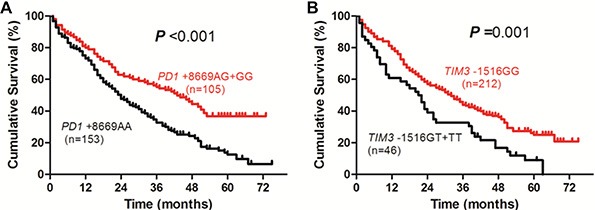
Overall survival curves of HBV-related HCC patients according to genotypes of PD1 +8669 A/G (A) or TIM3 −1516 G/T (B) polymorphisms estimated by Kaplan-Meier analysis and compared by the log-rank test

When all the 258 HBV-related HCC patients were included for the analysis of OS, Kaplan-Meier curve and log-rank test showed that patients with *PD1* +8669 G allele-containing genotypes (AG+GG) had significantly longer survival time than those with *PD1* +8669 genotype AA (*P* < 0.001, Figure 1A). The *TIM3* −1516 genotype GG was also significantly associated with longer survival time compared with *TIM3* −1516 T allele-containing genotypes (*P* = 0.001, Figure 1B). Higher albumin (ALB) and lower alpha-fetoprotein (AFP) levels, lower Child-Pugh score and lower TNM stage were also associated with longer OS (all *P* < 0.001, Supplementary Figure S1).

When those patients were included for the analysis of factors associated with OS, univariate analysis showed that total bilirubin (TBIL), ALB, AFP, Child-Pugh score, tumor size, TNM stage, *PD1* +8669 A/G and *TIM3* −1516 G/T polymorphisms were significantly associated with patients' survival (Table 2). In multivariate analysis, genotypes of *PD1* +8669 A/G (hazard ratio (HR), 1.835; 95% confidence interval (CI), 1.342–2.509; *P* < 0.001) and *TIM3* −1516 G/T (HR, 2.070; 95% CI, 1.428–3.002; *P* < 0.001) polymorphisms, together with gender (HR, 1.647; 95% CI, 1.013–2.676; *P* = 0.044), ALB (HR, 1.839; 95% CI, 1.306–2.588; *P* < 0.001), AFP (HR, 1.475; 95% CI, 1.073–2.027; *P* = 0.017), Child-Pugh score (HR, 1.735; 95% CI, 1.236–2.434; *P* = 0.001), and TNM stage (HR, 1.772; 95% CI, 1.277–2.459; *P* = 0.001), are significantly associated with the OS of the patients (Table 2).

**Table 2:** Univariate and multivariate analyses of factors associated with overall survival of the 258 HCC patients

	No. of patients	Overall survival (%)			Univariate analysis	Multivariate analysis	
		1 year	3 year	5 year	*P*	HR (95% CI)	*P*
Gender					0.581	1.647 (1.013–2.676)	0.044
Male	227	75.3	41.0	26.4			
Female	31	80.6	48.4	25.8			
Age (years)					0.246	1.256 (0.927–1.703)	0.142
≤ 50	136	80.1	46.3	27.9			
> 50	122	71.3	36.9	24.6			
HBV DNA (IU/mL)					0.156	1.326 (0.965–1.821)	0.081
≤ 10^4^	94	78.7	47.9	30.9			
> 10^4^	164	74.4	38.4	23.8			
ALT (IU/L)					0.109	0.928 (0.637–1.351)	0.697
≤ 40	111	82.9	46.8	30.6			
> 40	147	70.7	38.1	23.1			
AST (IU/L)					0.055	1.202 (0.785–1.842)	0.397
≤ 40	69	87.0	50.7	33.3			
> 40	189	72.0	38.6	23.8			
TBIL (μmol/L)					< 0.001	1.088 (0.787–1.504)	0.609
≤ 40	164	81.7	49.4	34.8			
> 40	94	66.0	28.7	11.7			
ALB (g/L)					< 0.001	1.839 (1.306–2.588)	< 0.001
≥ 35	120	87.5	59.2	44.2			
< 35	138	65.9	26.8	10.9			
AFP (ng/mL)					< 0.001	1.475 (1.073–2.027)	0.017
≥ 200	144	77.1	53.5	37.5			
≥ 200	114	74.6	27.2	12.3			
Child-Pugh score					< 0.001	1.735 (1.236–2.434)	0.001
A	164	85.4	50.0	34.8			
B + C	94	59.6	27.7	11.7			
Tumor size (cm)					< 0.001	1.030 (0.735–1.445)	0.862
< 5	172	77.3	50.6	33.1			
≥ 5	86	73.3	24.4	12.8			
TNM stage					< 0.001	1.772 (1.277–2.459)	0.001
I + II	156	77.6	51.9	37.8			
III + IV	102	73.5	26.5	8.8			
*PD1* + 8669					< 0.001	1.835 (1.342–2.509)	< 0.001
AG + GG	105	80.0	55.2	42.9			
AA	153	73.2	32.7	15.0			
*TIM3* −1516					0.001	2.070 (1.428–3.002)	< 0.001
GG	212	79.2	43.9	30.7			
GT +TT	46	60.9	32.6	6.5			

Abbreviations: HCC, hepatocellular carcinoma; HR, hazard ratio; CI, confidence interval; HBV, hepatitis B virus; ALT, alanine aminotransferase; AST, aspartate aminotransferase; TBIL, total bilirubin; ALB, albumin; AFP, alpha-fetoprotein.

### Association of *PD1* and *TIM3* polymorphisms with overall survival of HBV-related HCC patients according to treatments

**Figure 2:**
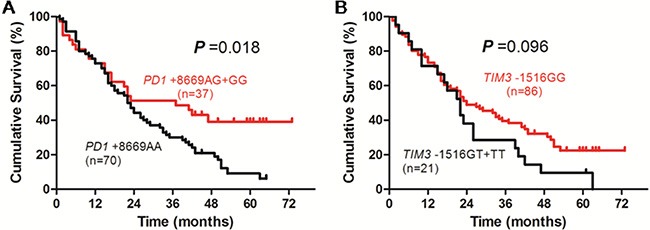
Overall survival curves of HBV-related HCC patients receiving surgical (resection or radiofrequency ablation) treatment according to genotypes of PD1 +8669 A/G (A) or TIM3 −1516 G/T (B) polymorphisms estimated by Kaplan-Meier analysis and compared by the log-rank test

In patients receiving surgical (tumor resection or radiofrequency ablation) treatment, the survival time of patients with *PD1* +8669 G allele-containing genotypes was significantly longer than those with *PD1* +8669 genotype AA (*P* = 0.018, Figure 2A). The survival time of patients with *TIM3* −1516 genotype GG was also longer than those with *TIM3* −1516 T allele-containing genotypes although the difference was not statistically significant (*P* = 0.096, Figure 2B). In addition, patients who had younger age and higher ALB and lower AFP levels also had longer OS (*P* = 0.036, *P* < 0.001 and *P* = 0.007, respectively, Supplementary Figure S2). Univariate analysis showed that age, TBIL, ALB, AFP, Child-Pugh score, TNM stage, *PD1* +8669 A/G and *TIM3* −1516 G/T polymorphisms were significantly associated with OS of the patients (Table 3). Multivariate analysis showed that *PD1* +8669 genotypes AG+GG (HR, 2.092; 95% CI, 1.218–3.593; *P* = 0.007) and *TIM3* −1516 genotype GG (HR, 2.370; 95% CI, 1.283–4.378; *P* = 0.006), together with younger age (HR, 2.358; 95% CI, 1.392–3.995; *P* = 0.001), higher ALB level (HR, 1.854; 95% CI, 1.117–3.076; *P* = 0.017), lower AFP level (HR, 1.793; 95% CI, 1.076–2.986; *P* = 0.025), were significantly associated with longer OS of the patients (Table 3).

**Table 3:** Univariate and multivariate analyses of factors associated with overall survival of the 107 HCC patients receiving surgical (tumor resection or radiofrequency ablation) treatment

	No. of patients	Overall Survival (%)			Univariate analysis	Multivariate analysis	
		1 year	3 year	5 year	*P*	HR (95% CI)	*P*
Gender					0.780	1.350 (0.646–2.818)	0.425
Male	92	72.8	37.0	22.8			
Female	15	73.3	40.0	20.0			
Age (years)					0.036	2.358 (1.392–3.995)	0.001
≤ 50	49	87.8	51.0	28.6			
> 50	58	60.3	25.9	17.2			
HBV DNA (IU/mL)					0.316	1.297 (0.789–2.129)	0.305
≤ 10^4^	37	73.0	43.2	27.0			
> 10^4^	70	72.9	34.3	20.0			
ALT (IU/L)					0.345	0.773 (0.423–1.414)	0.404
≤ 40	43	79.1	39.5	25.6			
> 40	64	68.8	35.9	20.3			
AST (IU/L)					0.149	1.087 (0.540–2.185)	0.816
≤ 40	26	80.8	46.2	30.8			
> 40	81	70.4	34.6	19.8			
TBIL (μmol/L)					0.003	1.196 (0.711–2.010)	0.500
≤ 40	69	78.3	44.9	30.4			
> 40	38	63.2	23.7	7.9			
ALB (g/L)					< 0.001	1.854 (1.117–3.076)	0.017
≥ 35	49	83.7	53.1	36.7			
< 35	58	63.8	24.1	10.3			
AFP (ng/mL)					0.007	1.793 (1.076–2.986)	0.025
< 200	58	74.1	50.0	34.5			
≥ 200	49	71.4	22.4	8.2			
Child-Pugh score					0.001	1.447 (0.815–2.569)	0.207
A	61	80.3	45.9	31.1			
B + C	46	63.0	26.1	10.9			
Tumor size (cm)					0.081	0.782 (0.450–1.359)	0.382
< 5	69	73.9	44.9	29.0			
≥ 5	38	71.1	23.7	10.5			
TNM stage					0.018	1.680 (0.981–2.877)	0.059
I + II	68	73.5	47.1	29.4			
III+IV	39	71.8	20.5	10.3			
*PD1* +8669					0.018	2.092 (1.218–3.593)	0.007
AG + GG	37	73.0	51.4	40.5			
AA	70	72.9	30.0	12.9			
*TIM3* −1516					0.096	2.370 (1.283–4.378)	0.006
GG	86	73.3	39.5	26.7			
GT + TT	21	71.4	28.6	4.8			

Abbreviations: HCC, hepatocellular carcinoma; HR, hazard ratio; CI, confidence interval; HBV, hepatitis B virus; ALT, alanine aminotransferase; AST, aspartate aminotransferase; TBIL, total bilirubin; ALB, albumin; AFP, alpha-fetoprotein.

**Figure 3:**
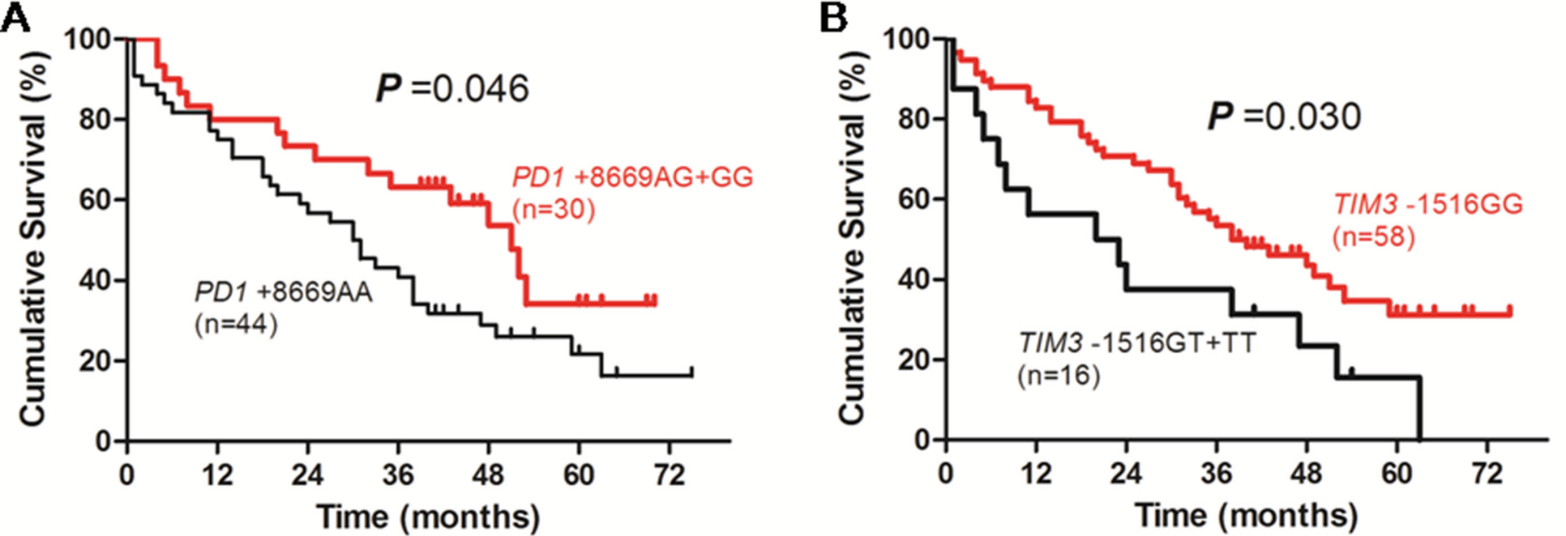
Overall survival curves of HBV-related HCC patients receiving transcatheter arterial chemoembolization according to genotypes of PD1 +8669 A/G (A) or TIM3 −1516 G/T (B) polymorphisms estimated by Kaplan-Meier analysis and compared by the log-rank test

In patients receiving TACE, the survival time of patients with *PD1* +8669 G allele-containing genotypes was significantly longer than those with *PD1* +8669 genotype AA (*P* = 0.046, Figure 3A). The survival time of patients with *TIM3* −1516 genotype GG was significantly longer than those with *TIM3* −1516 T allele-containing genotypes (*P* = 0.030, Figure 3B). Patients who had higher ALB level, lower Child-Pugh score and lower TNM stage also had longer OS (*P* < 0.001, *P* = 0.003 and *P* = 0.004, respectively, Supplementary Figure S3). Univariate analysis showed that ALB level, Child-Pugh score, tumor size, TNM stage, *PD1* +8669 A/G and *TIM3* −1516 G/T polymorphisms were associated with OS of the patients (Table 4). Multivariate analysis showed that *PD1* +8669 genotypes AG+GG (HR, 3.042; 95% CI, 1.470–6.294; *P* = 0.003) and *TIM3* −1516 genotype GG (HR, 2.055; 95% CI, 1.053–4.010; *P* = 0.035), together with higher ALB level (HR, 2.479; 95% CI, 1.083–5.672; *P* = 0.032), lower Child-Pugh score (HR, 2.701; 95% CI, 1.277–5.711; *P* = 0.009) and TNM stage (HR, 2.863; 95% CI, 1.034–7.930; *P* = 0.043), were significantly associated with longer OS of the patients (Table 4).

**Table 4:** Univariate and multivariate analyses of factors associated with overall survival of the 74 HCC patients receiving transcatheter arterial chemoembolization

	No. of patients	Overall Survival (%)			Univariate analysis	Multivariate analysis	
		1 year	3 year	5 year	*P*	HR (95%CI)	*P*
Gender					0.117	2.869 (0.796–10.343)	0.107
Male	66	87.5	75.0	28.8			
Female	8	75.8	47.0	62.5			
Age (years)					0.485	1.169 (0.584–2.340)	0.658
≤ 50	46	78.3	52.2	32.6			
> 50	28	75.0	46.4	32.1			
HBV DNA (IU/mL)					0.089	0.769 (0.408–1.447)	0.415
≤ 10^4^	24	75.0	41.7	20.8			
> 10^4^	50	78.0	54.0	38.0			
ALT (IU/L)					0.266	0.664 (0.278–1.588)	0.358
≤ 40	34	85.3	55.9	38.2			
> 40	40	70.0	45.0	27.5			
AST (IU/L)					0.247	2.273 (0.869–5.945)	0.094
≤ 40	18	94.4	55.6	44.4			
> 40	56	71.4	48.2	28.6			
TBIL (μmol/L)					0.072	1.491 (0.691–3.219)	0.309
≤ 40	54	81.5	55.6	37.0			
> 40	20	65.0	35.0	20.0			
ALB (g/L)					< 0.001	2.479 (1.083–5.672)	0.032
≥ 35	37	66.7	35.1	13.5			
< 35	37	86.5	64.9	51.4			
AFP (ng/mL)					0.137	0.740 (0.355–1.544)	0.423
< 200	45	80.0	53.3	42.2			
≥ 200	29	72.4	44.8	17.2			
Child-Pugh score					0.003	2.701 (1.277–5.711)	0.009
A	55	85.5	56.4	36.4			
B + C	19	52.6	31.6	21.1			
Tumor size (cm)					0.009	0.906 (0.303–2.709)	0.860
< 5	52	80.8	59.6	40.4			
≥ 5	22	68.2	27.3	13.6			
TNM stage					0.004	2.863 (1.034–7.930)	0.043
I + II	49	81.6	59.2	42.9			
III + IV	25	68.0	32.0	12.0			
*PD1* +8669					0.046	3.042 (1.470–6.294)	0.003
AG + GG	30	80.0	63.3	46.7			
AA	44	75.0	40.9	22.7			
*TIM3* −1516					0.030	2.055 (1.053–4.010)	0.035
GG	58	82.8	53.4	37.9			
GT + TT	16	56.2	37.5	12.5			

Abbreviations: HCC, hepatocellular carcinoma; HR, hazard ratio; CI, confidence interval; HBV, hepatitis B virus; ALT, alanine aminotransferase; AST, aspartate aminotransferase; TBIL, total bilirubin; ALB, albumin; AFP, alpha-fetoprotein.

**Figure 4:**
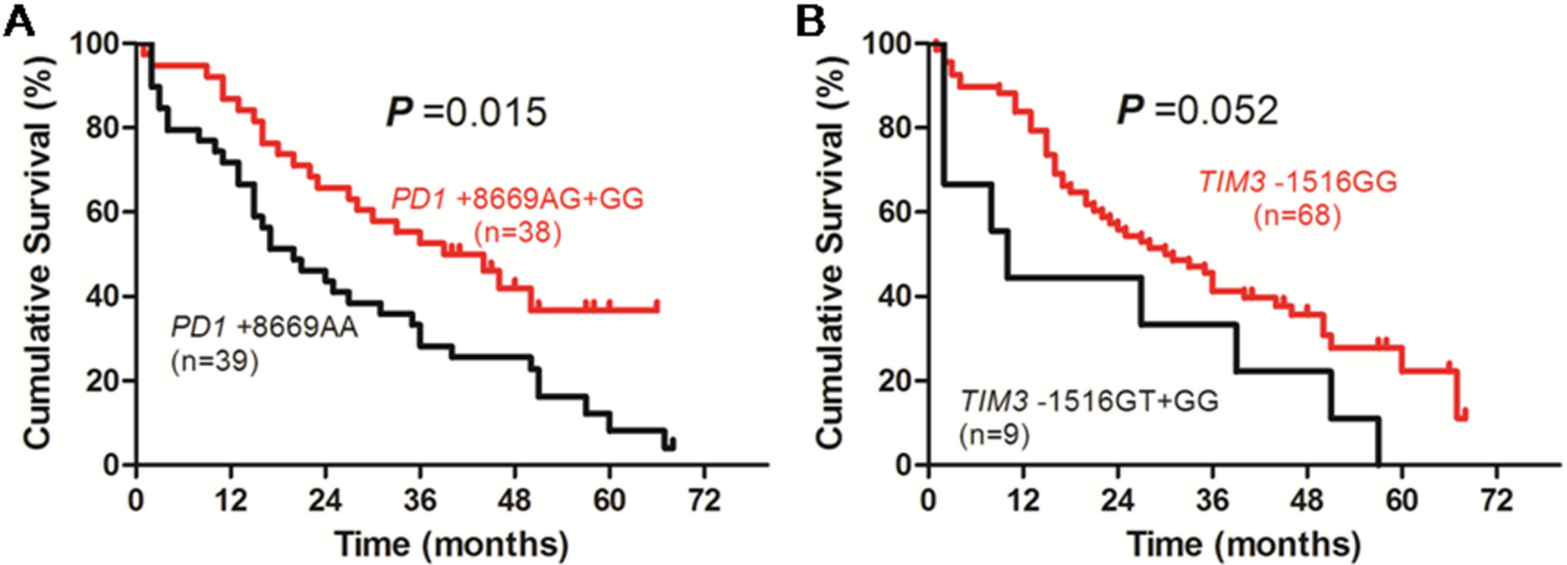
Overall survival curves of HBV-related HCC patients receiving supportive care and symptomatic treatment according to genotypes of PD1 +8669 A/G (A) or TIM3 −1516 G/T (B) polymorphisms estimated by Kaplan-Meier analysis and compared by the log-rank test

In patients receiving supportive and symptomatic treatment, the OS of patients with *PD1* +8669 G allele-containing genotypes was significantly longer than those with *PD1* +8669 genotype AA (*P* = 0.015, Figure 4A). The OS of patients with *TIM3* −1516 genotype GG was longer than those with *TIM3* −1516 T allele-containing genotypes although it was not statistically significant (*P* = 0.052, Figure 4B). Patients who had lower HBV DNA level, higher ALB level, lower Child-Pugh score and TNM stage also had longer OS (*P* = 0.001, *P* < 0.001, *P* = 0.002 and *P* = 0.008, respectively, Supplementary Figure S4). Univariate analysis showed that HBV DNA, TBIL, ALB and AFP levels, Child-Pugh score, TNM stage and *PD1* +8669 A/G polymorphism were associated with OS of the patients (Table 5). The *TIM3* −1516 G/T polymorphism was marginally associated with OS of the patients (*P* = 0.052, Table 5). Multivariate analysis showed that *PD1* +8669 genotypes AG+GG (HR, 1.909; 95% CI, 1.011–3.602; *P* = 0.046), together with lower HBV DNA level (HR, 1.984; 95% CI, 1.031–3.817; *P* = 0.040), higher ALB level (HR, 2.329; 95% CI, 1.156–4.692; *P* = 0.018), lower Child-Pugh score (HR, 2.064; 95% CI, 1.032–4.127; *P* = 0.040) and lower TNM stage (HR, 2.168; 95% CI, 1.174–4.003; *P* = 0.013), were significantly associated with longer OS of the patients (Table 5). The *TIM3* −1516 genotype GG was not significantly associated with OS of the patients (HR, 1.790; 95% CI, 0.744–4.308; *P* = 0.194, Table 5).

**Table 5:** Univariate and multivariate analyses of factors associated with overall survival of the 77 HCC patients receiving supportive and symptomatic treatment

	No. of patients	Overall Survival (%)			Univariate analysis	Multivariate analysis	
		1 year	3 year	5 year	*P*	HR (95% CI)	*P*
Gender					0.267	1.618 (0.621–4.217)	0.325
Male	69	78.3	40.6	29.0			
Female	8	87.5	37.5	0			
Age (years)					0.135	0.724 (0.388–1.351)	0.310
≤ 50	41	73.2	34.1	22.0			
> 50	36	86.1	47.2	30.6			
HBV DNA (IU/mL)					0.001	1.984 (1.031–3.817)	0.040
≤ 10^4^	33	87.9	57.6	42.4			
> 10^4^	44	72.7	27.3	13.6			
ALT (IU/L)					0.716	0.868 (0.404–1.865)	0.716
≤ 40	34	85.3	47.1	29.4			
> 40	43	74.4	34.9	23.3			
AST (IU/L)					0.570	1.659 (0.685–4.018)	0.262
≤ 40	25	88.0	52.0	28.0			
> 40	52	75.0	34.6	25.0			
TBIL (μmol/L)					0.037	0.682 (0.345–1.349)	0.271
≤ 40	41	87.8	48.8	39.0			
> 40	36	69.4	30.6	11.1			
ALB (g/L)					< 0.001	2.329 (1.156–4.692)	0.018
≥ 35	34	94.1	61.8	47.1			
< 35	43	67.4	23.3	9.3			
AFP (ng/mL)					0.017	1.444 (0.720–2.899)	0.301
< 200	41	78.0	58.5	36.6			
≥ 200	36	80.6	19.4	13.9			
Child-Pugh score					0.002	2.064 (1.032–4.127)	0.040
A	48	91.7	47.9	37.5			
B + C	29	58.6	27.6	6.9			
Tumor size (cm)					0.067	1.448 (0.756–2.772)	0.264
< 5	51	78.4	49.0	31.4			
≥ 5	26	80.8	23.1	15.4			
TNM stage					0.008	2.168 (1.174–4.003)	0.013
I + II	39	79.5	51.3	46.2			
III + IV	38	78.9	28.9	5.3			
*PD1* +8669					0.015	1.909 (1.011–3.602)	0.046
AG + GG	38	86.8	52.6	42.1			
AA	39	71.9	28.2	10.3			
*TIM3* −1516					0.052	1.790 (0.744–4.308)	0.194
GG	68	83.8	41.2	29.4			
GT + TT	9	44.4	33.3	0			

Abbreviations: HCC, hepatocellular carcinoma; HR, hazard ratio; CI, confidence interval; HBV, hepatitis B virus; ALT, alanine aminotransferase; AST, aspartate aminotransferase; TBIL, total bilirubin; ALB, albumin; AFP, alpha-fetoprotein.

## DISCUSSION

This prospective study found that *PD1* +8669 G/A and *TIM3* −1516 G/T polymorphisms were associated with the OS of HBV-related HCC patients. Specifically, *PD1* +8669 genotypes AG+GG were significantly associated with longer OS of the patients when the whole patients included in the final analysis or the patients according to treatments were analyzed. Furthermore, multivariate analysis in the whole patients included in the final analysis and in patients receiving different treatments confirmed that *PD1* +8669 A/G polymorphism was an independent factor associated with OS of the patients. For *TIM3* −1516 G/T polymorphisms, the genotype GG was significantly associated with longer OS when the whole patients included in the final analysis or patients receiving TACE were analyzed. In patients receiving surgical (tumor resection or radiofrequency ablation) treatment, multivariate analysis showed that *TIM3* −1516 genotype GG was also an independent factor significantly associated with longer OS of the patients. In patients receiving supportive and symptomatic treatment, *TIM3* −1516 genotype GG was not shown to be significantly associated with the OS of the patients possibly because of the relatively small number of patients in this subgroup.

The basis for the associations found in this study may relate to the recognized ability of PD-1 and TIM-3 to inhibit T-cell mediated antiviral and antitumor immunity as well as the functional relevance of these polymorphisms. PD-1 and TIM-3 overexpression in relation to T-cell dysfunction and exhaustion in chronic HBV infection and HCC development [5–9] and in predicting the outcome and the disease progression of HBV infection [10–12] and the prognosis of HCC [9, 13] have been demonstrated in many studies. Consistent with the associations found in this study, *PD1* +8669 genotypes GG and AG, which were related to longer OS of the HBV-related HCC patients, were associated with lower PD-1 mRNA levels [12] and the downregulation of PD-1 expression and upregulation of tumor necrosis factor (TNF)-α and interferon (IFN)-γ in patients with chronic HBV infection through interaction with miR-4717 [19]. For *TIM3* polymorphisms, the polymorphic variants of *TIM3* and the type of TIM-3-expressing cells including TIM-3-expressing dendritic cells, macrophages, and T cells were revealed to affect the recognition of apoptotic cells and the immune responsiveness [20]. The *TIM3* −1516 genotype GG, which was generally related to longer OS of the HBV-related HCC patients in this study, was suggestively associated with the downregulation of TIM-3 expression in chronic HBV infection [17] and lower tumor grade and less frequent lymph node metastasis in HBV-related HCC [17].

Blockade of immune checkpoint proteins including PD-1 and TIM-3 has been emerging as a promising therapeutic approach for various malignancies including HCC [21]. In patients with acute alcoholic hepatitis, blocking PD-1 and TIM-3 could reverse T-cell production of IFN-γ, reduce the numbers of interleukin 10-producing T cells, and increase antibacterial immune responses impaired in this condition [22].

Coexpression of PD-1 and TIM-3 was associated with more severe CD8 T cell exhaustion in mice with chronic viral infection or bearing solid tumors [23, 24]. Combined targeting of the PD-1 and TIM-3 pathways was revealed to be more effective in improving CD8 T cell responses and viral control in chronically viral infected mice [23] and in controlling tumor growth in solid tumor-bearing mice [24]. In colon carcinoma tumor-bearing mice, the combinatorial blockade of PD-1 and TIM-3 pathways was shown to be synergistic with a remarkable antitumor effect [25]. Therefore, combined blockade of PD-1 and TIM-3 pathways has shown very promising potential in the immunotherapy of chronic viral infection and tumors.

Blockade of immune checkpoint such as PD-1 or TIM-3 represents effective and promising immunotherapy for cancer. However, a lot of cancer patients failed to respond to the PD-1 pathway blockades. Furthermore, these new immunotherapies may generate so called immune-related adverse events (IRAEs) due to unbalancing the immune system [26]. Therefore, immunotherapy by immune checkpoint blockade is confronted with novel challenges in selecting patient populations, monitoring clinical responses, and predicting IRAEs [27]. Some parameters such as the overexpression of PD ligand 1 (PD-L1) and tumor-infiltrating immune cells and molecules are currently explored predictive biomarkers for the response to PD-1 pathway blockade. However, these biomarkers can not be used to accurately select patients for PD-1 pathway blockade due to the low prediction accuracy and dynamic changes [28, 29]. Based on the significant associations of *PD1* and *TIM3* polymorphisms with the OS of HBV-related HCC patients presented in this study, together with their role in chronic HBV infection and HBV-related HCC as well as the functional relevance to immune responses, it is reasonable to hypothesize that the determination of *PD1* +8669 A/G and *TIM3* −1516 G/T polymorphisms may be potential immunogenetic biomarkers for selecting appropriate patients, designing personalized strategy such as mono or dual checkpoint blockade and monitoring clinical responses, or even predicting IRAEs when the immunotherapy by blocking PD-1 and TIM-3 pathway, alone or dually, is administered clinically.

This study has some limitations. The sample size of patient population studied is relatively small. There is a lack of a validation patient population. The study population is limited to Chinese Han patients. The study did not analyze the association of the polymorphisms examined with the disease free survival (DFS) of the patients because of the inability to accurately obtain the DFS data in all the patients studied. All these limitations may compromise the validity of the findings. Therefore, replicative studies with larger sample size of populations with various ethnic backgrounds and accurately collected DFS and OS data are warranted to validate these findings.

In conclusion, this study, for the first time to our knowledge, showed that *PD1* +8669 A/G and *TIM3* −1516 G/T polymorphisms are associated with the OS of HBV-related HCC patients. These findings might have potential implications for selecting appropriate patients, designing personalised strategy of immunotherapy with checkpoint blockade, monitoring clinical responses, and predicting immune adverse events when the immunotherapy by blocking PD-1 and TIM-3 pathway, alone or dually, is clinically administered.

## MATERIALS AND METHODS

### Patients

A total of 262 HBV-related HCC patients were recruited from the First Affiliated Hospital of Xi'an Jiaotong University between January 1, 2009 to June 30, 2010. The diagnosis of HBV-related HCC was established based on the serological positivity for HBsAg, HBeAg or anti-HBe and anti-HBc for more than 6 months and either pathological examination of liver tissue of surgery or characteristics of HCC image of angiography, ultrasonography and computerized tomography (CT) and/or magnetic resonance imaging (MRI). The underlying cirrhosis of the patients was diagnosed according to the pathology of cirrhosis in liver tissue of surgery, or the presence of abnormal liver function, portal hypertension with ascites and esophageal varices, characteristics of liver cirrhosis image and splenomegaly on ultrasonography and/or CT and MRI [30]. The demographic and clinical data were collected in all the patients at the diagnosis of HCC. The tumor grade, size and metastasis were determined at diagnosis and might be confirmed in some cases after liver transplantation, tumor resection, radiofrequency ablation, or transcatheter arterial chemoembolization (TACE) for those who received corresponding treatment interventions. Patients with other severe diseases and comorbidities unrelated to HCC were excluded. All the patients included were adult of Han nationality and unrelated in kinship. The study was performed in accordance with Declaration of Helsinki and the protocol was approved by the Ethics Committee of the First Affiliated Hospital of Xi'an Jiaotong University. Informed consent was obtained from all the patients.

### Treatments and follow-up

Of the 262 HBV-related HCC patients, 4 patients (2 received liver transplantation and 2 received sorafenib) were excluded from the survival analysis because of the small number of patients in these treatments. The remaining 258 patients were included in the final analysis. Of these 258 patients, 52 patients underwent tumor resection, 55 patients received radiofrequency ablation, 74 patients received TACE and 77 patients received supportive and symptomatic treatment. Because of the relatively small number of patients and the comparable overall survival between hepatic resection and radiofrequency ablation in early stage HCC [31, 32], we combined the patients who underwent tumor resection and radiofrequency ablation as 1 group of treatment, surgical treatment (107 patients), in the analysis. The demographic and clinical data in the patients were obtained at the establishment of HBV-related HCC diagnosis. The data of therapy were recorded in each patient and the survival data of the patients were prospectively collected until June 30, 2015.

### Genotyping of *PD1* and *TIM3* polymorphisms

Genomic DNA was extracted from whole blood using TIANamp Genomic DNA Kit (Tiangen Biotech (Beijing) Co., Ltd., China) according to the manufacturer's instruction. Genotyping of *PD1* +8669 G/A (rs10204525) and *TIM3* −1516 G/T (rs10053538) polymorphisms was carried out by bidirectional polymerase chain reaction (PCR) amplification of specific alleles (Bi-PASA) and PCR-restriction fragment length polymorphism, respectively, as described elsewhere [16, 17].

### Statistical analysis

Statistical analyses were carried out using a SPSS16.0 software (SPSS Inc., Chicago, IL, USA). Data were expressed as the median (range). Survival duration of the patients was defined as the period from the date of first diagnosis of HCC to the date of death (clinical endpoint) or the date of last follow-up (censored). Age, gender, HBV DNA, alanine aminotransferase (ALT), aspartate aminotransferase (AST), TBIL, ALB, Child-Pugh grade, tumor size, TNM stage and AFP at HCC diagnosis and genotypes of *PD1* +8669 G/A and *TIM3* −1516 G/T polymorphisms were included as candidate factors for the OS of the patients. Overall survival differences in the patients were analyzed by Kaplan-Meier curve and compared by log-rank test. Cox regression was used to perform multiple analyses to identify factors associated with the OS of the patients and to estimate the HR and its 95% CI. The statistical tests were two-sided and the significance was set at *P* value < 0.05.

## SUPPLEMENTARY MATERIALS FIGURES


