# Supplementary material for: Genetic polymorphisms of immune checkpoint proteins PD-1 and TIM-3 are associated with survival of patients with hepatitis B virus-related hepatocellular carcinoma
Source: Oncotarget. 2016 Mar 28;7(18):26168–80. doi: 10.18632/oncotarget.8435 (PMC5041972; doi:10.18632/oncotarget.8435)
Supplement: Supplementary file 1 [file oncotarget-07-26168-s001.pdf]

Genetic polymorphisms of immune checkpoint proteins PD-1 and TIM-3 are associated with survival of patients with hepatitis B virus-related hepatocellular carcinoma

Supplementary Materials

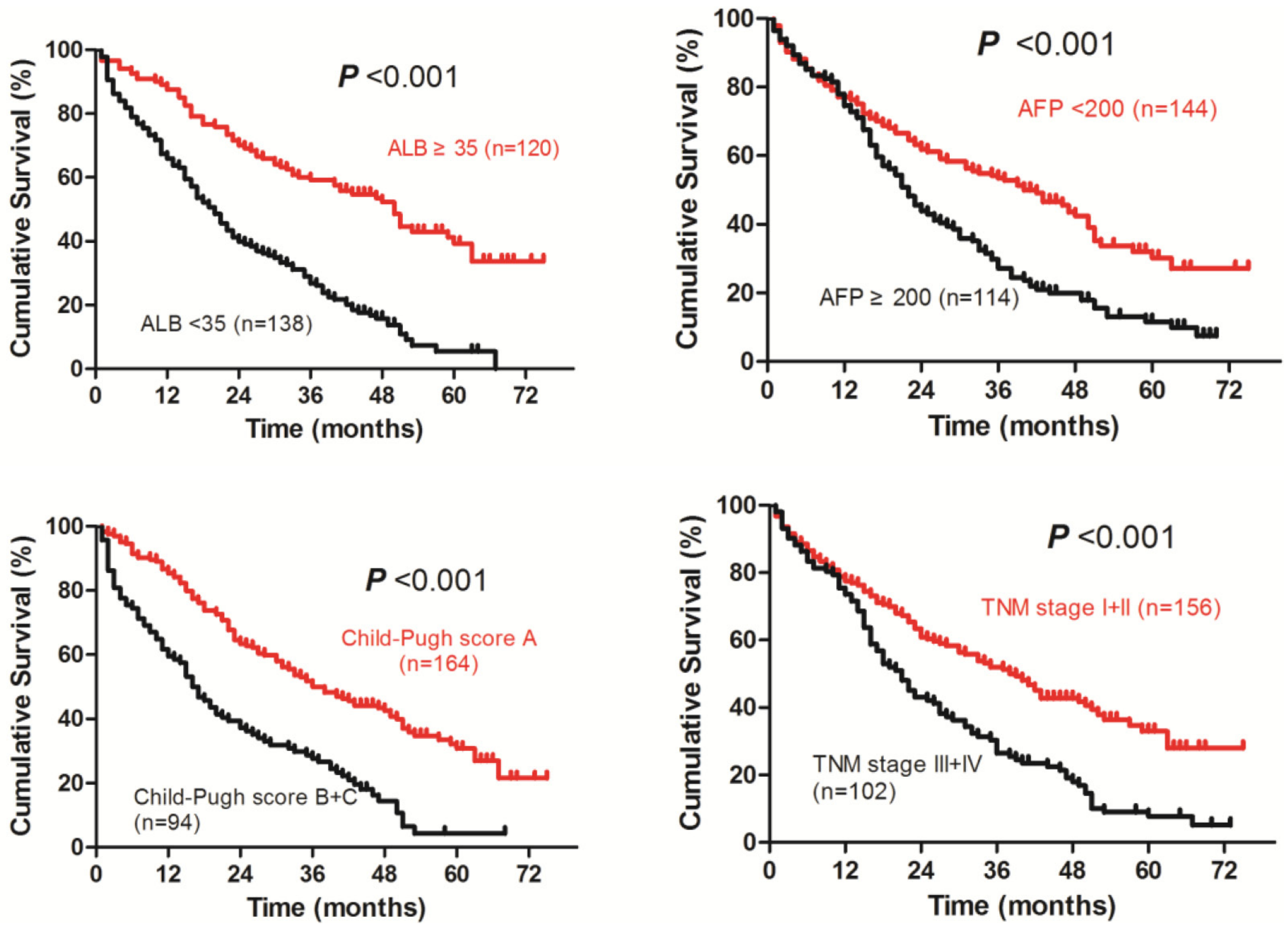

Supplementary Figure S1: Overall survival curves of the 258 HBV-related HCC patients according to ALB, AFP, Child-Pugh score and TNM stage estimated by Kaplan–Meier analysis and compared by the log–rank test.

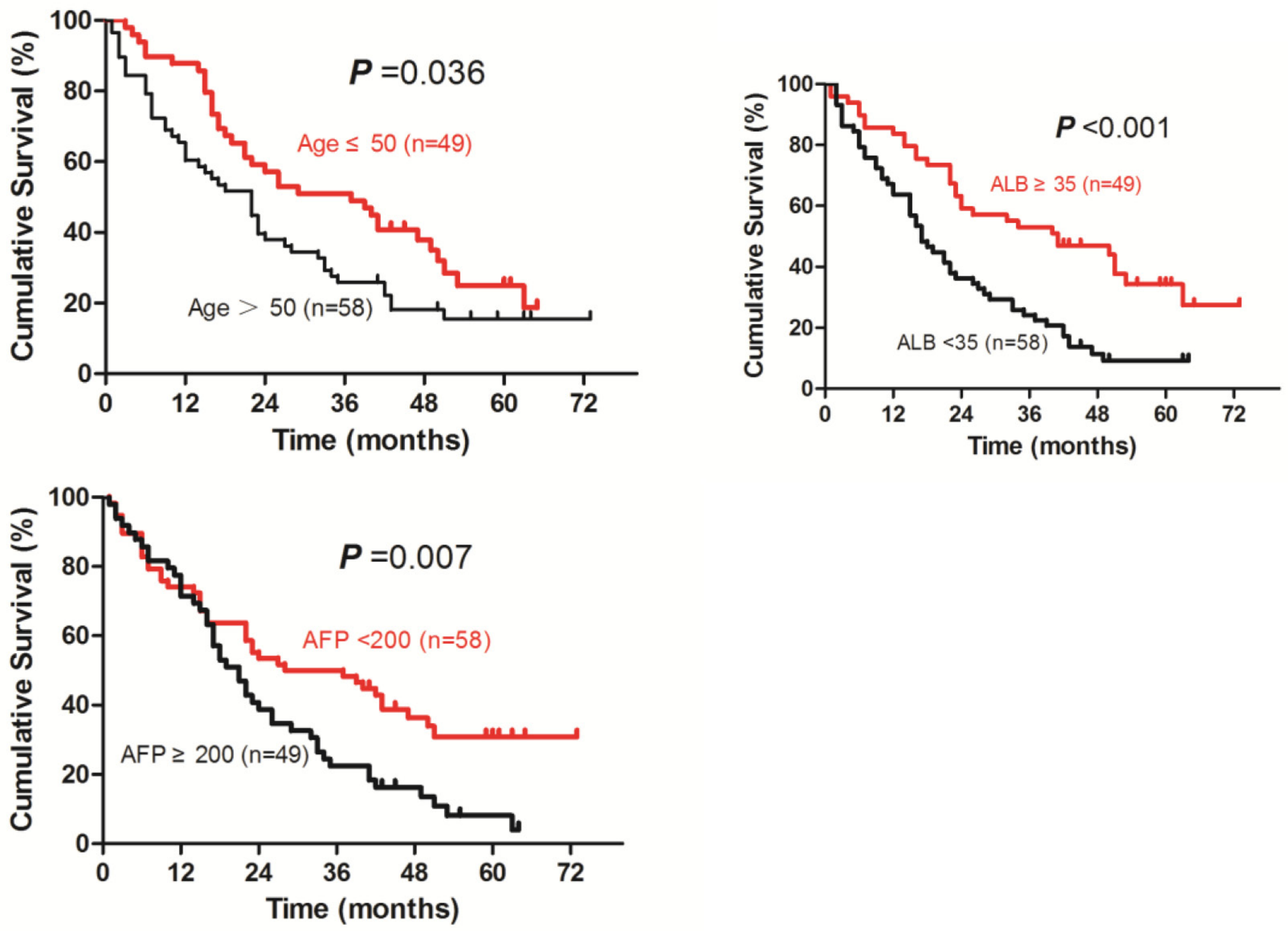

Supplementary Figure S2: Overall survival curves of the 107 HBV-related HCC patients receiving surgical (resection or radiofrequency) treatment according to age, ALB and AFP estimated by Kaplan–Meier analysis and compared by the log–rank test.

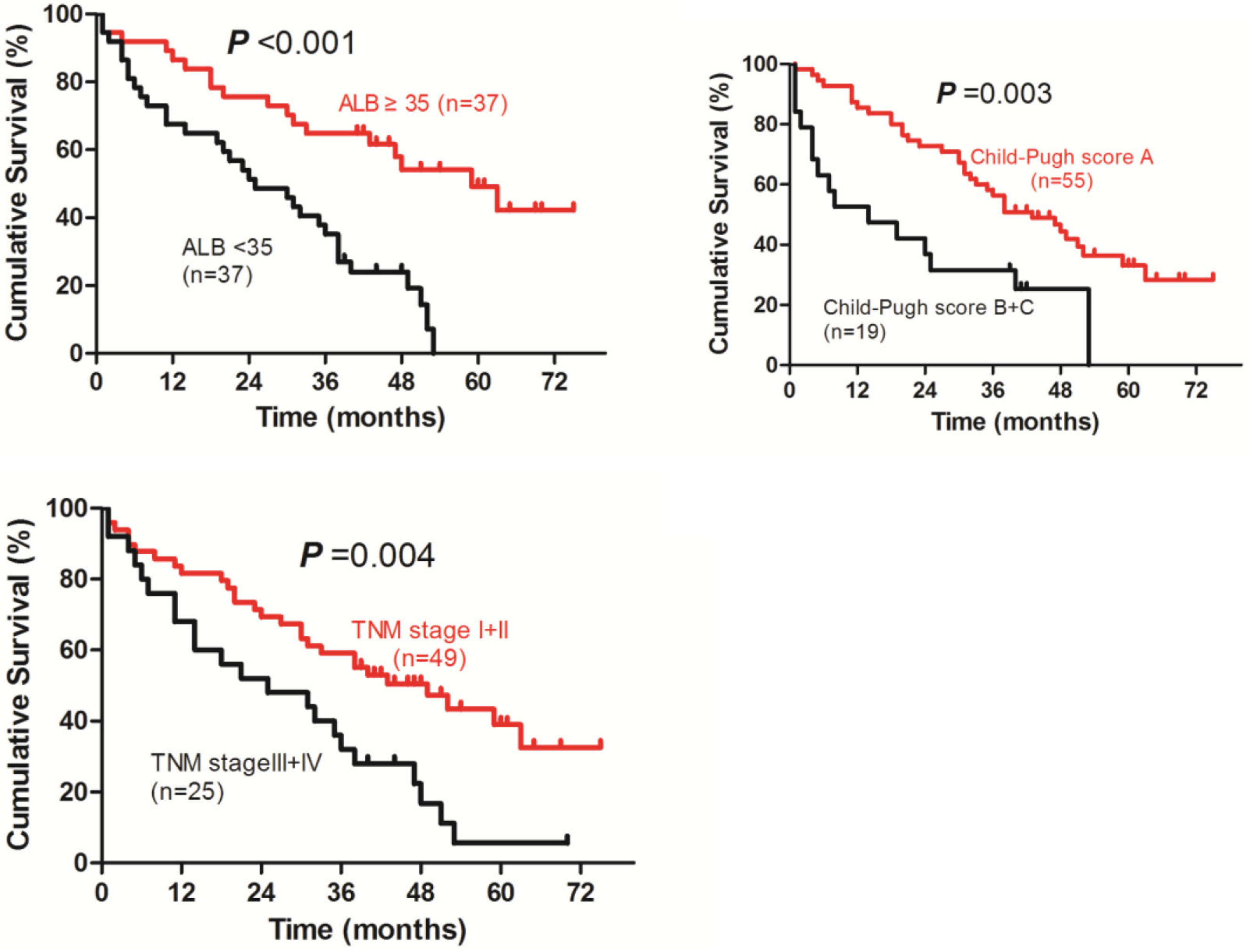

Supplementary Figure S3: Overall survival curves of the 74 HBV-related HCC patients receiving transcatheter arterial chemoembolization according to ALB, Child-Pugh score and TNM stage estimated by Kaplan–Meier analysis and compared by the log–rank test.

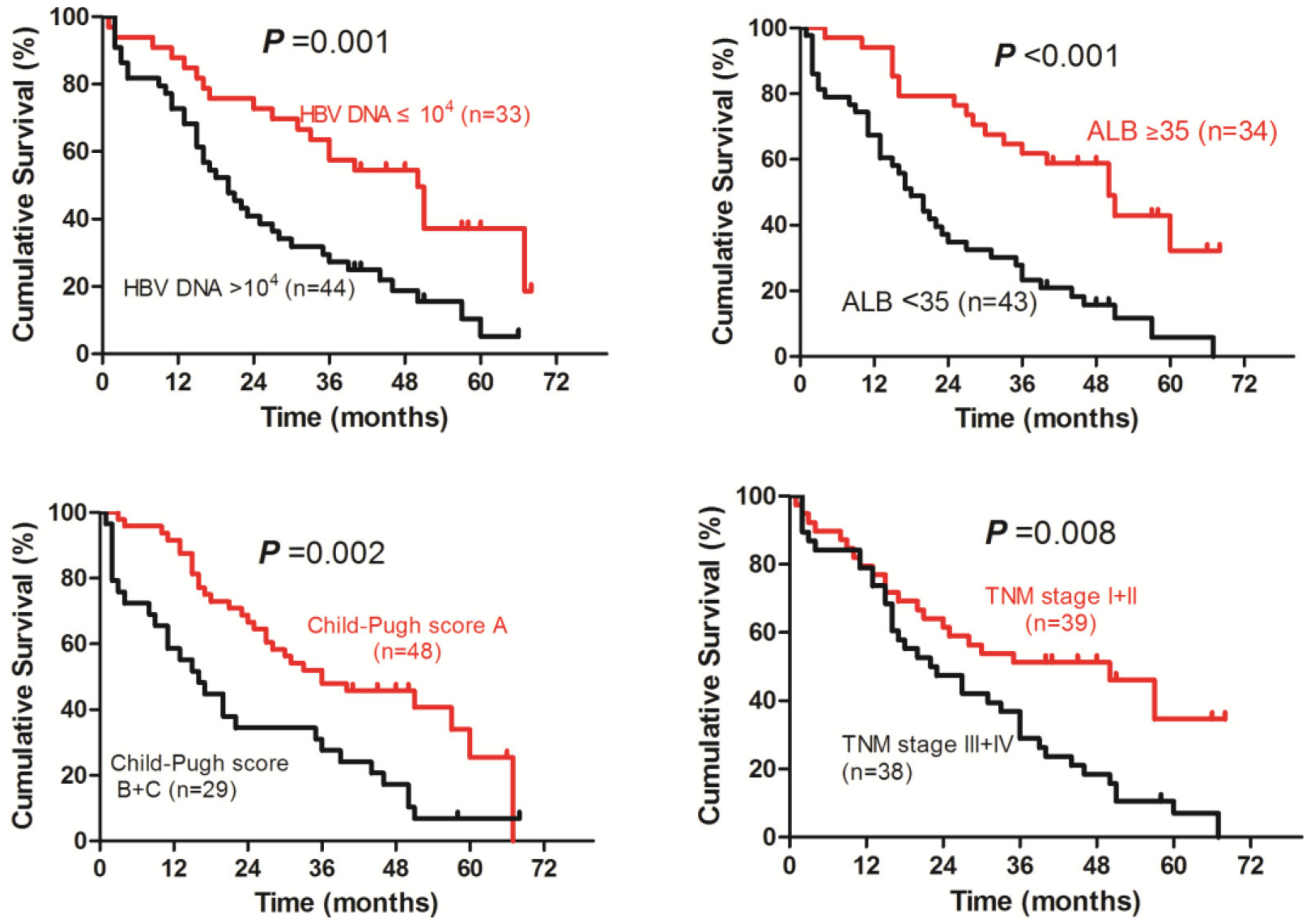

Supplementary Figure S4: Overall survival curves of the 77 HBV-related HCC patients receiving supportive and symptomatic treatment according to HBV DNA, ALB, Child-Pugh score and TNM stage estimated by Kaplan–Meier analysis and compared by the log–rank test.
